# Analysis of T-DNA integration and generative segregation in transgenic winter triticale (*x Triticosecale* Wittmack)

**DOI:** 10.1186/1471-2229-12-171

**Published:** 2012-09-25

**Authors:** Goetz Hensel, Sylwia Oleszczuk, Diaa Eldin S Daghma, Janusz Zimny, Michael Melzer, Jochen Kumlehn

**Affiliations:** 1 Leibniz Institute of Plant Genetics and Crop Plant Research (IPK), Plant Reproductive Biology, Corrensstr. 3, 06466, Gatersleben, Germany; 2 Plant Breeding and Acclimatization Institute, National Research Institute, Radzików, 05-870, Błonie, Poland; 3 Leibniz Institute of Plant Genetics and Crop Plant Research (IPK), Structural Cell Biology, Corrensstr. 3, 06466, Gatersleben, Germany; 4 National Gene Bank and Genetic Resources, Agriculture Research Center, 12619, Giza, Egypt

**Keywords:** *Agrobacterium*, Winter triticale (*x Triticosecale* Wittmack), Sexual transmission, Transgene expression, *gfp*, Copy number

## Abstract

**Background:**

While the genetic transformation of the major cereal crops has become relatively routine, to date only a few reports were published on transgenic triticale, and robust data on T-DNA integration and segregation have not been available in this species.

**Results:**

Here, we present a comprehensive analysis of stable transgenic winter triticale cv. Bogo carrying the selectable marker gene *HYGROMYCIN PHOSPHOTRANSFERASE* (*HPT*) and a synthetic *green fluorescent protein* gene (*gfp)*. Progeny of four independent transgenic plants were comprehensively investigated with regard to the number of integrated T-DNA copies, the number of plant genomic integration loci, the integrity and functionality of individual T-DNA copies, as well as the segregation of transgenes in T_1_ and T_2_ generations, which also enabled us to identify homozygous transgenic lines. The truncation of some integrated T-DNAs at their left end along with the occurrence of independent segregation of multiple T-DNAs unintendedly resulted in a single-copy segregant that is selectable marker-free and homozygous for the *gfp* gene. The heritable expression of *gfp* driven by the maize *UBI-1* promoter was demonstrated by confocal laser scanning microscopy.

**Conclusions:**

The used transformation method is a valuable tool for the genetic engineering of triticale. Here we show that comprehensive molecular analyses are required for the correct interpretation of phenotypic data collected from the transgenic plants.

## Background

Triticale, the artificial wheat x rye amphiploid, was created in an attempt to combine the grain quality and productivity of wheat with the superior performance of rye in marginal environments. Since the development of the early hybrids, substantial breeding progress has been made by conventional means. Nevertheless, the global cropping area devoted to triticale remains low, and the bulk of its production is concentrated in central and eastern Europe [1]. Given the prevailing climatic conditions in this region, >90% of the crop is represented by winter (vernalization-requiring) cultivars. Its major end-uses are as feed or fodder, but improvements in its grain milling and bread making quality could allow an extension to its use in human consumption or as an industrial feedstock. A particularly attractive prospect lies in its use as a source of bioenergy, but generating the necessary significant changes to the plant to allow this will probably require genetic modification. Hence there is a need to develop robust protocols for its genetic transformation.

The genetic engineering of triticale is still in its infancy. The direct delivery of DNA into protoplasts has achieved transient transgene expression, but not stable integration [2,3]. Difficulties in regenerating plants from isolated protoplasts have prompted the exploration of more readily regenerable explant materials, among which the immature embryo has proven to be the most promising [4-7]. The earliest reported stable transgenic triticale plants relied on the biolistic treatment of immature embryo scutella [8]. A similar approach targeting haploid embryo-like structures resulted in only the transient expression of the transgene and no adult plants were regenerated [9]. A first applicative approach was published by Doshi et al. (2007) who used the embryo-specific *LTP1* promoter to show the effect of the *C1* and *Bperu* maize genes on anthocyanin biosynthesis in triticale [10].

*Agrobacterium*–mediated gene transfer has proven to be an effective means of transforming each of the major cereal species, including triticale [11-13]. For the latter however, robust data on T-DNA integration and segregation were not provided thus far. Using plants produced by means of a previously published protocol [13], we here provide comprehensive information on transgenic triticale in terms of T-DNA integration, copy number, integrity and inheritance. Moreover, we provide evidence of consistent transgene expression across generations.

## Results

### Primary transgenic plants

**Figure 1:**
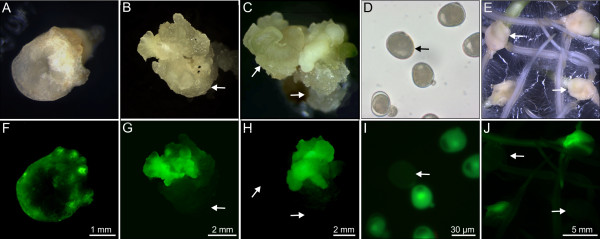
**GFP accumulation in transgenic lines as shown by correlative microscopy using white light (A-E) and excitation by far blue light (F-J). **Immature embryos (**A**, **F**) three days, (**B**, **G**) three weeks and (**C**, **H**) six weeks after co-culture with *Agrobacterium*; (**D**, **I**) immature pollen formed by the T_0_ plant TG5E02 isolated from anthers, collected in 0.4 M mannitol and examined under a fluorescence microscope; (**E**, **J**) immature T_1_ embryos from TG5E03 were germinated on K4N medium and their roots examined 5 days after embryo isolation. Non-transgenic tissue domains and segregants are indicated by arrows in **B**, **C**, **D** and **E** and their fluorescence microscopic counterparts **G**, **H**, **I** and **J**, where no or only background fluorescence is seen in these positions. Note that the ratios of transgenic and non-transgenic individuals displayed in **I** and **J** are not representative.

Primary transgenic triticale plants that we had reported on in a previous article presenting protocols for several small grain cereals [13] were subjected in the present study to a comprehensive analysis. In this context, we also provide some complementary information on the elaboration of the transformation protocol previously published. As shown in Table 1, a two or five day period of pre-culturing (prior to exposure to *A. tumefaciens*) was required to obtain transgenic plants. Moreover, inoculation with *Agrobacterium* proved successful provided the immature embryos were stacked on filter paper moistened with liquid co-cultivation medium, rather than being submerged in the medium. Table 1 further shows, that transgenic plants were exclusively obtained when barley co-culture medium (BCCM) was used, whereas wheat co-culture medium (WCCM) failed to give rise to transgenics under the same conditions. Likewise, a hypervirulent derivative of *A. tumefaciens* strain LBA4404 resulted in the delivery of transgenic regenerants, whereas AGL-1 surprisingly did not do so under the conditions tested. The expression of *gfp* was monitored throughout the transformation process (Figure 1A-J). After three days of co-cultivation with *A. tumefaciens*, fluorescing foci were visible at the margin of scutella of the immature embryos. After a further 3–6 weeks, GFP accumulation was particularly strong in the rapidly growing portion of the callus (Figure 1F-H), whereas emerging leaves and roots showed fluorescence of lower intensity. The transgenic regenerants confirmed by a PCR-based amplification of a *gfp* fragment did not show any obvious morphological effect associated with transgenicity.

**Table 1:** **Effect of pre-cultivation, *A. tumefaciens *strain and co-cultivation medium on the generation of stable transgenic triticale**

**Strain**	**Pre-culture time and co-culture conditions**	**Transgenic lines per 100 IEs**
**AGL-1/ pYF133**	0 d, BCCM	-
0 d, WCCM		-
	5 d, BCCM	-
	5 d, WCCM	-
	2 d, BCCM, filter paper	-
	5 d, BCCM, filter paper	-
	5 d, WCCM, filter paper	-
**LBA4404/ pSB187**	0 d, BCCM	-
0 d, WCCM		-
	5 d, BCCM	-
	5 d, WCCM	-
	2 d, BCCM, filter paper	3.6
	5 d, BCCM, filter paper	4.0
	5 d, WCCM, filter paper	-

A set of 50 IEs was processed per treatment. BCCM - barley co-culture medium, WCCM - wheat co-culture medium [13], IEs - immature embryos.

### Number, integrity and generative transmission of integrated T-DNAs

**Figure 2:**
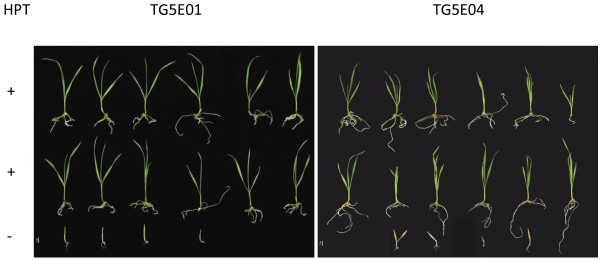
**Rescue of segregating T**_**1**_**immature embryos from two of the four primary transgenics (TG5E01 and TG5E04).** Seedling reaction to hygromycin was assessed after germination on hygromycin-containing K4N media for ten days. Hygromycin-resistant plantlets were considered as HPT positive (+), whereas susceptible ones were classified as HPT negative (−). HPT: hygromycin phosphotransferase (conferring hygromycin resistance).

Pollen produced by each of the four primary transgenic plants segregated with respect to *gfp* expression (Figure 1I). When embryos formed by the primary transgenic plants were germinated on a medium containing hygromycin, *gfp* expression was strong in both the scutellum and the emerging root, with a particularly high level in the root tip (Figure 1J). The selective medium discriminated between transgene carriers and non-carriers, with the former developing normally and the latter no longer able to grow after the emergence of the coleoptile, and unable to synthesize chlorophyll (Figure 2). The observed segregation ratio was consistent in three of the four cases with the presence of a single T-DNA insertion locus, while the progeny of the primary transgenic TG5E03 included significantly fewer transgene carriers than expected from a monogenic segregation (Table 2).

**Table 2:** **T**_**1 **_**segregation for hygromycin resistance**

**T**_**0**_** plants**	**T-DNA copies (DNA gel blot)**	**T**_**1**_** plants analyzed**	**Hygromycin resistant/ susceptible plants**	**Segregation ratio**	**Χ**^**2**^** value as to expected ratio of 3:1**	**Likelihood (P) according to Χ**^**2**^** test**
**TG5E01**	3	73	59:14	4.2:1	1.8	>0.18
**TG5E02**	1	92	74:18	4.1:1	3.5	>0.05
**TG5E03**	2	96	54:42	1.3:1	24.8	<0.01
**TG5E04**	2	98	76:22	3.5:1	4.5	>0.05

Deviation from monogenic segregation (as shown by χ^2^ test) was only significant among the progeny of TG5E03.

**Figure 3:**
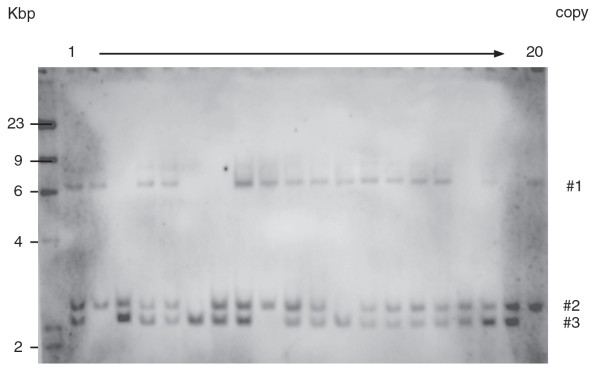
**DNA gel blot analysis of TG5E01 T**_**1**_**segregants. **Genomic DNA was digested by *Hin*dIII, gel separated and transferred onto a Hybond-N membrane and hybridized against a DIG labeled fragment of *gfp*. The copy numbers were designated according to Tables 3 and 4.

**Figure 4:**
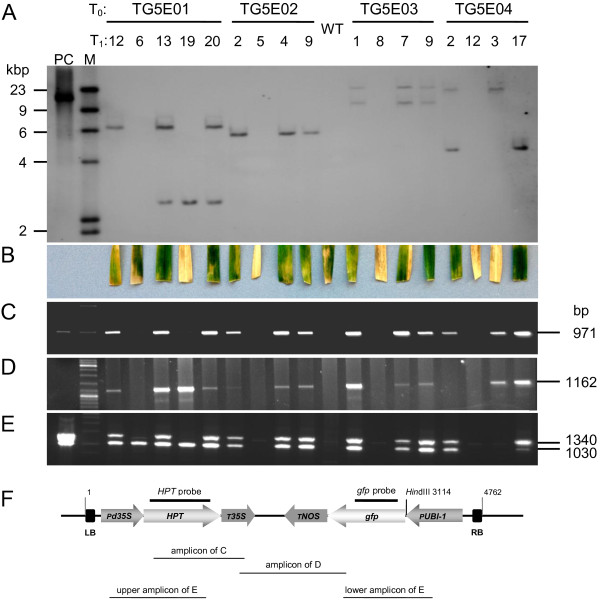
**Analysis of transgene events. **(**A**) DNA extracted from a set of T_1_ plants of each event subjected to DNA gel blotting, and probed with a DIG labeled fragment of *HPT.* Note that copy #3 of TG5E01 as revealed and indicated in Figure 3 was not highlighted by the *HPT* probe used here. (**B**) Detached leaf hygromycin assay 10 days after transfer to hygromycin-containing medium. (**C**) Amplification of the *HPT* coding region and the *CaMV 35S* terminator. (**D**) Amplification of the *gfp* coding region and the *CaMV 35S* terminator. (**E**) Duplex PCR targeting the *CaMV 35S* and the *UBI-1* promoter, as well as the *HPT* and *gfp* coding sequence. (**F**) The T-DNA based transformation cassette. LB – left border, P*35S* – promoter of the *CaMV 35S* promoter, *HPT* – *HYGROMYCIN PHOSPHOTRANSFERASE* gene, T*35S* – terminator of the *CaMV 35S* coding sequence. T*NOS* – terminator of the *NOPALINE SYNTHASE* gene, *gfp* – synthetic *green fluorescent protein* gene (S65T), P*UBI-int* – maize *UBI-1* promoter with first intron, RB – right border. The full length of the T-DNA, the relative position of the *Hin*dIII restriction site as well as the positions of probes used in A (*HPT* probe) and Figure 3 (*gfp* probe) as well as amplicons shown in **C**, **D** and **E** are indicated.

In order to comprehensively analyse the independent transgenic events obtained, T_1_ siblings were subjected to DNA gel blot analysis successively using *HPT* and *gfp* probes in order to characterize the integration sites. The *gfp*-profile of progeny of TG5E01 is given as an example in Figure 3. The *HPT* profiles shown in Figure 4A indicated that the number of T-DNA copies present in TG5E01, TG5E02, TG5E03 and TG5E04 was, respectively, two, one, two and two, while the *gfp* probe also highlighted a third copy in TG5E01 (Figure 3). The analysis also showed that all three T-DNA copies present in TG5E01 and both copies in TG5E04 segregated independently of one another, while the two copies in TG5E03 co-segregated (Figure 3 and 4A). A complete T-DNA has the size of 4762 bp. In the case of complete T-DNAs, the digestion of genomic DNA using *Hin*dIII is expected to result in hybridizing fragments larger than 3114 bp (Figure 4F), as was seen in all plants except the descendants from TG5E01, where the two fragments carrying copies #2 and #3 were smaller.

The derived structure of the transgenes in TG5E02 and TG5E03 was confirmed by PCR assays based on primer pairs spanning various parts of the two transgenes (Figure 4C-F); however, for TG5E01, the PCR analysis of T_1_ plant 6 carrying only the T-DNA copy #3 (see Figure 3, plant lane 6) suggested that the *HPT* expression cassette was absent (Figure 4C,D,E, plant 6). Likewise, T-DNA copy #2, which was present in TG5E01 T_1_ plant 19 in addition to copy #3, did not show amplicons in Figure 4C and upper E either, indicating that it was also truncated with respect to the *HPT* cassette*.* Another example of incomplete T-DNA was found in TG5E04, where the single copy present in T_1_ plant 3 was truncated concerning the *HPT* expression cassette as revealed by PCR analysis (Figure 4E, upper band). Moreover, the also missing lower band in Figure 4E suggests another deletion in the promoter-*gfp* junction, whereas the DNA gel blot analysis had demonstrated the presence of both the *HPT* and the *gfp* sequences in this plant, and the internal *HPT* and *gfp* sequences were both successfully amplified.

### Transgene expression in the T_1_ and T_2_ generation

When T_1_ plant leaf segments were challenged with hygromycin, those carrying a functional copy of *HPT* remained green, whereas those lacking the transgene or a functional version became bleached (Figure 4B). In all cases, the outcome of the test concurred with the conclusions drawn from the PCR and/or DNA gel blot assays (Figure 4A-E). Note the intermediate reaction of the non-transformed control (Figure 4B), which suggested that this assay on its own is insufficiently diagnostic of the incorporation of a *HPT* transgene.

**Figure 5:**
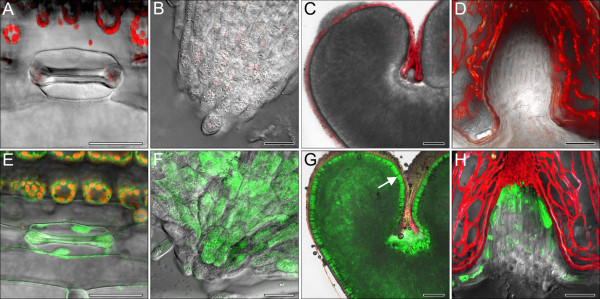
**Localization of GFP accumulation using confocal laser scanning microscopy. **Chlorenchyma cells and stomatal cells in the lower leaf epidermis of (**A**) non-transformed and (**E**) transgenic plants. (**B**) Non-transformed and (**F**) transgenic root tip. Transverse section through a mature (**C**) non-transformed and (**G**) transformed caryopsis, the latter showing a low level of GFP activity in the endosperm and a high level in the aleurone layer (arrow). Detail of a transverse section through a mature grain showing the nucellar projection in a (**D**) non-transformed and (**H**) transgenic plant. Very strong GFP activity was observed at the base and the lateral periphery of the nucellar projection. Chlorophyll or grain husk autofluorescence shown in red. Bars indicate 50 μm.

GFP was detectable in the root, leaf and mature caryopsis of plants carrying the single copy transgene (TG5E02*)* (Figure 5A-H). In particular, the GFP signal was largely confined to cytosol. The level of *gfp* expression varied considerably between cell types. In the leaf, it was highest in the chlorenchyma and the stomatal guard cells (Figure 5A,E). In the mature caryopsis, the most intense signal was present in the aleurone layer (Figure 5C, G) and in the nucellar projection (Figure 5D, H).

**Figure 6:**
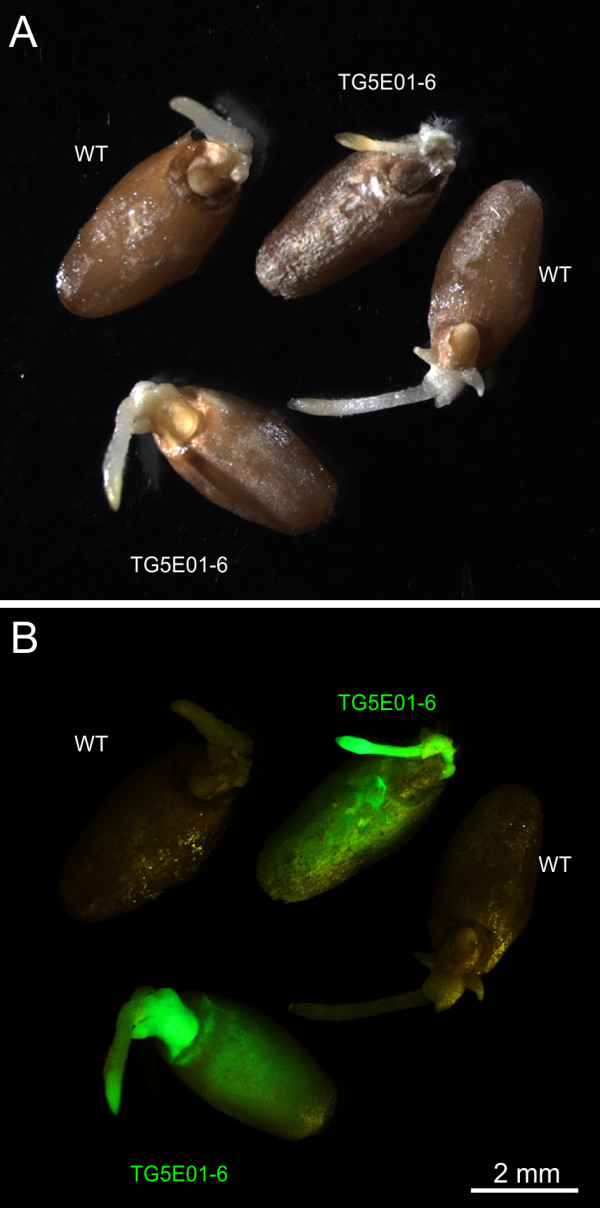
**Reporter gene expression in T**_**2**_**grains after seven days of germination. **(**A**) Illumination by white light and (**B**) excitation with far blue light; transgenic (TG5E01-6) and wild type (WT) control grains are indicated.

Twelve T_2_ populations and control descendants from an azygous T_1_ segregant were tested for the expression of *gfp* in the root tip (Table 3). As expected, some of these families segregated (TG5E01-12, TG5E03-1, TG5E03-7 and TG5E04-2), but it was possible to identify five non-segregating (presumably transgene-homozygous) plants. All TG5E01-6 T_2_ individuals accumulated GFP (Table 3, Figure 6) although they only contain a truncated T-DNA fragment (for DNA gel blot see Figure 3 plant lane 6, for T-DNA truncation see Figure 4C-F). In this case, T-DNA truncation along with independent segregation of multiple T-DNAs unintendedly resulted in a selectable marker-free segregant that is homozygous with regard to the *gfp* gene. By contrast, none of the TG5E04-3 T_2_ individuals tested expressed *gfp,* thereby confirming the missing PCR-fragment of the *gfp* expression cassette from the respective T_1_ plant as shown in Figure 4E (lower band). Given the outcome of the leaf hygromycin test and the missing PCR fragments in the T_1_ plant TG5E04-3 (Figure 4B,E and F), the conclusion drawn was that the transgene copy present in this plant comprised non-functional sequences with respect to both transgenes. All data regarding the integrity, functionality and segregation of the T-DNA copies are summarized in Table 4. With regard to the gene-of-interest (*gfp*), five out of eight independent transgene copies proved functional, while the presumed functionality of two further copies could not unequivocally be shown due to the presence of other T-DNAs. In all those cases, the observed phenotype could be explained by genotypic data of the plants.

**Table 3:** **Transgene segregation in twelve T**_**2 **_**populations, based on *gfp *expression in seedling tissue**

**T**_**1 **_**plants**	**T-DNA copies (DNA gel blot)**	**T**_**2 **_**plants analyzed**	**Segregation of*gfp*expression**	**Segregation ratio observed/ (presumed for hemizygosity)**	**Zygosity concluded**
**TG5E01-6**	1 (#3)	25	25:0	25:0 (3:1)	homozygous
**TG5E01-12**	2 (#1 + #3)	22	18:4	4.5:1 (15:1)	hemizygous
**TG5E01-19**	2 (#2 + #3)	25	25:0	25:0 (15:1)	homozygous^n.s.^
**TG5E01-20**	2 (#1 + #2)	28	28:0	28:0 (15:1)	homozygous ^n.s.^
**TG5E02-2**	1	22	22:0	22:0 (3:1)	homozygous *
**TG5E02-4**	1	17	17:0	17:0 (3:1)	homozygous*
**TG5E02-9**	1	20	20:0	20:0 (3:1)	homozygous*
**TG5E03-1**	2 (#1 + #2)	21	7:14	1:2 (3:1)	hemizygous
**TG5E03-7**	2 (#1 + #2)	20	4:16	1:4 (3:1)	hemizygous
**TG5E03-8**	0	20	0:20	0:20	azygous
**TG5E04-2**	2 (#1 + #2)	18	14:4	3.5:1 (15:1)	hemizygous
**TG5E04-3**	1 (#1)	17	0:17	0:17 (3:1)	unascertainable^1^
**TG5E04-12**	0	14	0:14	0:14	azygous

*) Significant, n.s.: not significant, according to χ^2^ test.

1) TG5E04-3 carries a non-functional copy of *gfp*.

**Table 4:** The integrity, functionality and segregation of the T-DNA copies present in four primary transgenics

**T**_**0**_	**T**_**1**_									**T**_**2**_
**T-DNA**	**PCR**				**DNA gel blot**				**Phenotype**	**Phenotype**
	***P35S-HPT***	***HPT-T35S***	***PUBI1-gfp***	***gfp-TNOS***	***HPT***	***gfp***	***gfp*segregation**	**integration loci**	**hygromycin**^**R **^**(leaf assay)**	**GFP (seedlings)**
**TG5E01**										
copy #1	+	+	+	+	+	+	14:6 (2.3:1)	3	+	+ ^b^
copy #2	-	-	n.d. ^a^	+	+	+	18:2 (9.0:1)		-	
copy #3	-	-	+	-	-	+	17:3 (5.6:1)		-	+
**TG5E02**										
copy #1	+	+	+	+	+	+	18:2 (9.0:1)	1	+	+
**TG5E03**										
copy #1	+ ^b^	+ ^b^	+ ^b^	+ ^b^	+	+	3:17 (0.2:1)	1^c^	+ ^b^	+ ^b^
copy #2					+	+				
**TG5E04**										
copy #1	-	+	-	+	+	+	11:8 (1.4:1)	2	-	-
copy #2	+	+	+	+	+	+	12:7 (1.7:1)		+	+

a) Not determined, because an analysis independent of any of the two other copies was impossible.

b) Positive for at least one of the two transgene copies, however, these copies could not be analysed independent of one another.

c) Single case of this study where two T-DNA copies were co-integrated in one chromosomal locus.

## Discussion

Our initial aim was to develop a robust protocol for the stable genetic transformation of winter triticale. The approach taken rested heavily on transformation protocols established for its parental species, wheat and rye. The amenability of immature embryos to co-cultivation with *A. tumefaciens* in liquid culture was trialed, as this provides an efficient means of processing immature embryos in barley [14], and the same approach is effective in rye [15]. Unfortunately, however, it does not seem to work well in wheat (unpublished data), where desiccation of the immature embryos appears to improve the transformation efficiency [16]. In practice, triticale behaved like wheat in this respect, as transformation was only obtained when the immature embryos were co-cultivated on filter paper soaked with co-culture medium (Table 1). A similar study focusing on the spring triticale cultivar ‘Wanad’ compared the effectiveness of the three selectable marker genes *BAR*, *HPT* and *NPTII* driven by one of maize *UBI-1*, cauliflower mosaic virus *35S* or *A. tumefaciens NOS* promoter, respectively [12], and concluded that the best combination was *NOS::NPTII,* even though *NOS* performs poorly in a monocotyledonous host [17]. In the present study, the *HPT* selectable marker gene was preferred, a gene which has also proven useful e.g. in barley [18,19], wheat and maize [13]. The *gfp* reporter gene was an efficient tool for monitoring transgenesis and the subsequent expression of the transgene (Figure 1F-J) [13,20].

Two of the three multiple T-DNA insertion events involved independent integration sites. In barley, >50% of multiple transgenic events induced by agro-infection involved only one integration site [21]. A more comprehensive analysis of transformation outcomes has been made in *Arabidopsis thaliana*, where the number of T-DNA copies integrated at a single site appears to be dependent not only on the identity of the *A. tumefaciens* strain and the explant, but also on the transformation methodology as well as the origin of replication of the vector providing the T-DNA [22]. The occurrence of transformation events with multiple T-DNA copies being integrated in independent genomic loci of triticale opens up the opportunity to generate transgenic segregants with reduced copy number. Moreover, co-introduction of effector and selectable marker gene using two different T-DNAs may give rise to selectable marker-free transgenics after independent segregation of the loci in the T_1_. While a similar case unintendedly occurred in the present study (TG5E01 T_1_ plant 6), a directed approach using barley has recently been presented by Kapusi et al. [23]. Although *Agrobacterium*-mediated transformation generally results in the less frequent integration of truncated transgenes than biolistic transfer, as many as 44% of primary wheat transgenics have been shown to carry incomplete T-DNAs [24] with many involving truncations at the left T-DNA border [25]. In barley, meanwhile, only 3% (of 260 primary transgenics analysed) retained the full T-DNA [26]. Truncation of the T-DNA can be expected to result in a loss of transgene function, as was indeed the case in the present experiments that revealed truncations in 37.5% of the integrated T-DNAs analysed.

The non-Mendelian segregation of transgenes among T_1_ progeny is a commonplace observation, and several hypotheses have been promoted to explain this phenomenon, such as T_0_ chimerism, multiple independently assorting insertion loci and transgene silencing induced by multiple transgene copies or DNA rearrangements [24,27-29]. In some cases, false positives can arise due to the expression of non-incorporated transgene cassettes including those carried by persisting *Agrobacterium*[30]. Non-Mendelian transgene segregation has been noted in triticale [12], but since this observation was based on a histochemical reporter gene assay and did not include any DNA analysis, its basis could not be ascertained. In the present study, fewer transgenic progeny was obtained than expected in the case of TG5E03, which suggests this plant to be chimeric with regard to transgenicity. This interpretation is corroborated by DNA gel blot, PCR and leaf assay, which indicated that all functional elements be present in at least one of the two coupled T-DNA copies (Figure 4A-F). Nonetheless, one of the copies may have produced aberrant mRNA causing post-transcriptional gene silencing. However, the non-Mendelian segregation observed in the T_2_ families derived from TG5E03 is anticipated to be caused solely by transgene silencing in some siblings, because chimerism can generally be ruled out in generations later than T_0_.

As monitored using confocal laser scanning microscopy, *gfp* expression was widely distributed, but concentrated in the cytosol (Figure 5). This localization mirrors what has been observed in transgenic barley and wheat [31], where the level of reporter gene expression in the aleurone and the endosperm was comparable to that driven by either the barley bi-functional *α-AMYLASE/SUBTILISIN INHIBITOR* (*ISA*) or the wheat *EARLY-MATURING* (*EM*) promoter [31,32].

## Conclusion

The transformation method used in the present study is a valuable tool for the genetic engineering of triticale. The availability of reliable transformation technology should encourage the application of current functional genomics technologies to triticale, and accelerate the biotechnological-based approach to its improvement. In the present study, we show that comprehensive molecular analyses are required for the correct interpretation of phenotypic data collected from transgenic plants.

## Methods

### Plant material

Grains of the winter triticale (*x Triticosecale* Wittmack) cultivar ‘Bogo’ were germinated at 14/12°C day/night under a 12 h photoperiod with a photon flux density during the light period of 136 μmol s^-1^ m^-2^. After three weeks, the seedlings were vernalized by cultivating for eight weeks at 4°C under an 8 h photoperiod, re-potted and then grown in a glasshouse (18/16°C day/night, 16 h photoperiod, 170 μmol s^-1^ m^-2^ photon flux density).

### Choice of *A*. *tumefaciens* strain

**Figure 7:**
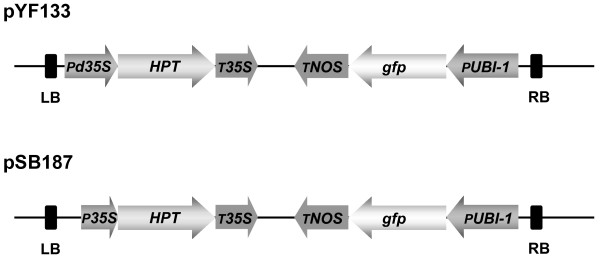
**Schematic representation of T-DNA regions of the binary vectors used for transformation. **LB - left border; *Pd35S* - *CaMV double 35S promoter*; *P35S* - *CaMV 35S promoter*; *HPT* - *HYGROMYCIN PHOSPHOTRANSFERASE* selectable marker gene conferring resistance to hygromycin; *T35S* - *CaMV 35S* terminator; *TNOS* - *Agrobacterium NOPALINE SYNTHASE* terminator; *gfp* - *green fluorescent protein* gene; *PUBI-1* - maize *UBIQUITIN-1* promoter; RB - right border.

The hypervirulent *A. tumefaciens* strain AGL-1 was initially used for infection. The strain harbors the binary vector pYF133 [33] which provides *CaMV d35S::HPT* as a selectable marker gene, a synthetic *gfp* sequence [34] driven by the maize *UBI-1* promoter [35] as the reporter gene, and the pCAMBIA vector backbone [36] (Figure 7). The T-DNA borders are derived from a nopaline-producing Ti plasmid. Later experiments used a hypervirulent derivative of strain LBA4404 [37], which harbors the binary vector pSB187; this differs from pYF133 in the promoter chosen to drive *HPT* (*CaMV 35S*) and the vector backbone which was derived from pLH6000 [38] (Figure 7). Both pYF133 and pSB187 were introduced into *A. tumefaciens* via electroporation.

### Isolation of immature embryos and their pre-culture and co-cultivation with *A. tumefaciens*

Immature caryopses were harvested 12–16 days post pollination, bathed for 3 min in 70% ethanol, further disinfected by immersion in 5% w/v sodium hypochlorite, 0.1% v/v Tween 20 for 15 min and washed five times in sterile, distilled water. Immature embryos were excised and plated scutellum side up on pre-cultivation medium as described elsewhere [13]. A frozen glycerol stock of *A. tumefaciens* (200 μL sampled from a culture with an OD_600_ of 2.0 suspended in 200 μL 15% v/v glycerol) was seeded into 10 mL antibiotic-free CPY medium and cultured overnight at 28°C with shaking. Prior to inoculation, the pre-cultivated immature embryos were transferred into liquid co-cultivation medium (BCCM) [13], which was then immediately replaced by 600 μL *A. tumefaciens* culture. After vacuum infiltration (1 min at 500 mbar), the immature embryos were washed with BCCM. The pre-cultured and inoculated immature embryos were then either stacked on filter paper disks moistened with 300 μL BCCM or WCCM or incubated in 2.5 mL of the same media. The co-cultivation of explants and *Agrobacterium* took place in the dark at 21°C for 48–72 h without agitation. A total of 50 immature embryos divided into 5 sets comprising 10 embryos each were used per treatment.

### Tissue culture

After co-cultivation, the immature embryos were transferred to BCIM [13] containing 150 mg·L^-1^ timentin to kill any remaining *A. tumefaciens*. Calli formed over the following two weeks were transferred to BCIM containing 25 mg L^-1^ hygromycin and 150 mg L^-1^ timentin for a further two weeks and then removed to K4N regeneration medium [39] containing 25 mg L^-1^ hygromycin and 150 mg L^-1^ timentin. The cultivation plates were exposed to 136 μmol s^-1^ m^-2^ photon flux density over a 16 h photoperiod. Regenerating shoots were transferred to glass tubes containing the same medium until the plantlets were vigorous enough to be potted into soil.

### DNA analysis

The PCR template comprised genomic DNA extracted from ~100 mg snap-frozen leaf using DNAzol (Invitrogen, Karlsruhe, Germany), according to the manufacturer’s instructions. PCRs were based on the amplification of 100 ng template primed by the sequences listed in Table 5. Amplicons were separated by agarose gel electrophoresis and visualized by Ethidium bromide staining (Figure 4). Plants testing positive with the PCR assays were subjected to DNA gel blot analysis to characterize the integration site(s) and transgene copy number. At least 25 μg genomic DNA, extracted as described in [40], was digested with *Hin*dIII, separated by agarose gel electrophoresis and blotted onto a Hybond N membrane. A gene-specific probe (*gfp* or *HPT*) was labeled with DIG as recommended by the supplier (Roche, Mannheim, Germany).

**Table 5:** Primer sequences used for the PCR analysis of putative transgenic regenerants

**Primer**	**Sequence 5′ – 3′**
35S-F2-C	CATGGTGGAGCACGACACTCTC
35S-term-C	CATGAGCGAAACCCTATAAGAACCC
GH-35S-term-F1	AATCACCAGTCTCTCTCTAC
GH-Hyg-F1	GATCGGACGATTGCGTCGCA
GH-Hyg-R2	TATCGGCACTTTGCATCGGC
GH-Ubi-F3	CCGTTCCGCAGACGGGATCGATCTAGGATAGGTA
GH-GFP-F1	GGTCACGAACTCCAGCAGGA
GH-GFP-R2	TACGGCAAGCTGACCCTGAA

### Embryo rescue and segregation analysis of hygromycin resistance

T_1_ caryopses were harvested 21–28 days after pollination and the immature embryos were placed for 24 h on Gamborg’s B5 medium [41] to induce germination. They were then transferred on to K4N medium [39] supplemented with 100 mg L^-1^ hygromycin, and held for up to ten days at 24°C under a 16 h photoperiod.

### Hygromycin leaf assay

*HPT* expression in leaf segments of putative transgenic plants was analyzed following [42], attaching the leaf segments by their base to MS culture medium [43] containing 200 mg L^-1^ hygromycin and 0.05% v/v Tween 20. The plates were incubated for 7–10 days at 24°C under a 16 h photoperiod.

### Microscopic analyses

Florets were harvested prior to anthesis and pollen grains released into 0.4 M mannitol. The expression of *gfp* was analysed using an Axiovert 200M fluorescence microscope equipped with the 38HE filter set (Zeiss, Oberkochen, Germany). Leaf, root and mature grain samples were analyzed with a LSM 510 META confocal laser scanning microscope (Zeiss, Jena, Germany) using a 488 nm laser line for excitation. GFP signals were detected with a 505–530 nm band pass filter. Autofluorescence was recorded with a 650 nm long-pass filter.

## Competing interests

The author(s) declare that they have no competing interests.

## Authors’ contributions

GH designed the transformation experiments, participated in the design and analysis of the molecular studies and drafted the manuscript. SO performed the transformation experiments and participated in drafting the manuscript. DD performed the microscopical studies. JZ and MM participated in the design and organisation of the study and helped to draft the manuscript. JK conceived of the study, and participated in its design and organisation and finalized the manuscript. All authors read and approved the final manuscript.
